# Social Drivers of Health and Firearm Storage Practices

**DOI:** 10.1001/jamanetworkopen.2025.13280

**Published:** 2025-06-02

**Authors:** Tarang Parekh, Annaliese Pena, Meghana Bhaskar, Jee Won Park

**Affiliations:** 1 Department of Epidemiology, College of Health Sciences, University of Delaware, Newark

## Abstract

**Importance:**

Understanding the primary social drivers of unsafe firearm storage practices is essential for preventing firearm-related injuries.

**Objective:**

To examine household firearm ownership and storage practices, assess their association with social drivers of health (SDOH), and identify factors associated with unsafe storage practices among adult residents.

**Design, Setting, and Participants:**

This cross-sectional analysis used 2022 Behavioral Risk Factor Surveillance System data from adults in 5 US states. Data analysis occurred from April 1 to October 2, 2024.

**Exposures:**

Participation in the Behavioral Risk Factor Surveillance System. SDOH include food insecurity, housing insecurity, social isolation, job loss or employment insecurity, feeling stressed, transportation barriers, and financial hardship.

**Main Outcomes and Measures:**

Firearm ownership and storage were assessed using structured questions. Storage practices were categorized as safest (all firearms unloaded), intermediate risk (at least 1 loaded and locked), and unsafe (at least 1 loaded and unlocked). Multivariable weighted logistic regression models estimated adjusted odds ratios (aORs) for firearm ownership and storage practices, accounting for demographic, socioeconomic, behavioral and mental health characteristics, and state firearm policies.

**Results:**

The unweighted study included 44 736 adults. The weighted population was 52.5% (95% CI, 51.5%-53.5%) female; 1.6% (95% CI, 1.4%-1.9%) American Indian, Alaska Native, and Pacific Islander; 10.0% (95% CI, 9.2%-10.8%) Asian non-Hispanic; 7.7% (95% CI, 7.2%-8.3%) Black non-Hispanic; 25.1% (95% CI, 24.1%-26.1%) Hispanic; 52.5% (95% CI, 51.5%-53.5%) White non-Hispanic; and 3.1% (95% CI, 2.7%-3.6%) multiracial. Firearms were owned in 29.3% of households (17 146 respondents), with 67.9% (11 396 respondents) storing them unloaded, 15.9% (2391 respondents) storing them loaded and locked, and 16.4% (2816 respondents) storing them loaded and unlocked. Unsafe storage was most common among non-Hispanic Black firearm owners (aOR, 2.23; 95% CI, 1.39-3.57). Food insecurity (aOR, 3.09; 95% CI, 1.29-7.40), housing insecurity (aOR, 1.66; 95% CI, 1.01-2.79), transportation barriers (aOR, 2.16; 95% CI, 1.19-3.90), and financial hardship (aOR, 2.22; 95% CI, 1.16-4.28) were significantly associated with unsafe storage practices. Households with children had higher odds of firearm ownership (aOR, 1.22; 95% CI, 1.05-1.43), but lower odds of unsafe storage (aOR, 0.38; 95% CI, 0.26-0.55). Child access prevention laws were associated with lower odds of firearm ownership (aOR, 0.57; 95% CI, 0.51-0.63) and unsafe storage (aOR, 0.52; 95% CI, 0.40-0.68). Behavioral risks like binge alcohol use (aOR, 1.39; 95% CI, 1.01-1.93) and life dissatisfaction (aOR, 1.81; 95% CI, 1.05-3.11) were associated with increased firearm ownership and unsafe storage.

**Conclusions and Relevance:**

In this cross-sectional study of US adults, firearm ownership was more prevalent among non-Hispanic White, higher-income, and highly educated households, but unsafe storage was more common among non-Hispanic Black and lower-income households. SDOH and high-risk behaviors, including alcohol misuse and life dissatisfaction, were linked to unsafe storage. Targeted public health initiatives are crucial for improving firearm safety.

## Key Points

**Question:**

What is the association between firearm ownership, storage practice, and social drivers of health in US adults?

**Findings:**

In this cross-sectional study of 44 736 US adults, 29.3% were in firearm-owning households, of whom 16.4% reported unsafe storage (loaded and unlocked), which was more common among non-Hispanic Black households despite ownership being most prevalent among non-Hispanic White households. Social drivers of health, including food and housing insecurity, transportation barriers, and financial hardship, were significantly associated with more than double the likelihood of unsafe firearm storage.

**Meaning:**

The connection between unsafe firearm storage and social factors highlights the importance of interventions targeting behaviors, economic inequalities, and systemic inequities.

## Teaser

This cross-sectional study examines household firearm ownership and storage practices, assesses their association with social drivers of health, and identifies factors associated with unsafe firearm storage practices.

## Introduction

Firearm-related violence is a critical public health issue in the US, accounting for approximately 50 000 deaths annually.^1^ The Second Amendment of the US Constitution uniquely guarantees the right to own firearms, shaping both laws and cultural attitudes, although its interpretation remains debated.^2,3,4^ As a result, guns are widely available, and efforts to regulate them face strong opposition. In 2022, firearm violence caused over 48 000 deaths in the US, an average of 132 per day.^5^ The presence of firearms in the home is a critical risk factor for suicide,^6^ particularly for adolescents, who are more likely to use firearms in suicidal acts, increasing the lethality of suicidal behaviors.^7,8,9^ Case-control studies consistently demonstrate a robust association between household firearm ownership and suicide deaths, particularly among adolescents.^10,11^ Moreover, regions with higher firearm ownership tend to exhibit elevated suicide rates, underscoring the direct impact of firearm availability on suicide risk.^10,11^

Understanding the primary drivers of unsafe firearm storage practices is essential for preventing firearm-related injuries. Although psychiatric factors, such as substance use disorders, mood disorders (eg, major depression), and a history of suicidal behavior, contribute to suicide risk, firearm injuries, and related deaths,^8,12^ the accessibility of firearms, especially when stored unsafely, can amplify these risks.^13,14^ Histories of violent behavior, exposure to violence, and hazardous substance use are also associated with firearm injuries.

Addressing the social drivers of firearm injuries requires a multidisciplinary approach. The World Health Organization defines social drivers or determinants of health (SDOH) as conditions shaping people’s lives, including poverty, income inequality, and food insecurity, which correlate with higher rates of violent crime.^1,15,16,17^ Although there is no research directly linking SDOH to firearm storage practices, some evidence highlights their role in firearm injuries. For instance, Smith et al^15^ found that even when controlling for other factors, food insecurity alone was associated with an increased risk of firearm-related injuries. A recent national survey of firearm owners highlighted financial barriers as one of the primary considerations in choosing a firearm-locking device for safe storage.^18^ Communities that are underresourced and characterized by high poverty, limited economic opportunities, and low social mobility tend to experience elevated rates of violence.^1,19^ These social conditions often stem from entrenched structural inequities, frequently tied to systemic racism, which may further elevate the risk of gun violence.

To date, to our knowledge, no studies have comprehensively examined and compared key SDOH in relation to firearm ownership and storage practices. This study seeks to address this gap and aims to (1) characterize adult residents by household firearm ownership and storage practices, (2) analyze the association between household firearm storage practices and SDOH, and (3) identify factors associated with ownership, as well as intermediate and/or unsafe storage practices.

## Methods

This cross-sectional study analyzed 2022 Behavioral Risk Factor Surveillance System (BRFSS) data, focusing on the optional firearm storage modules in 5 states, and the Social Determinants of Health and Equity module, which was implemented in nearly one-half of the states, including all 5 states that participated in the firearm storage module. Therefore, our analysis was restricted to these 5 states—California, Minnesota, Nevada, New Mexico, and Ohio—yielding a sample of 62 990 adults.^20^ The BRFSS is an annual, state-based, nationally representative telephone survey conducted by the Centers for Disease Control and Prevention (CDC) across all 50 US states, the District of Columbia, Puerto Rico, the US Virgin Islands, and Guam. It targets noninstitutionalized adults aged 18 years and older, excluding individuals residing in institutions such as nursing homes, prisons, and correctional facilities. Data are anonymized and publicly available, which allows for secondary analysis without institutional review board review or informed consent, in accordance with 45 CFR §46. Detailed methods are on the CDC website.^21^ We followed the Strengthening the Reporting of Observational Studies in Epidemiology (STROBE) reporting guidelines for cross-sectional studies.

### Firearm Storage and Ownership

The BRFSS firearm module has 3 sequential questions to assess firearm ownership and storage practices. First, participants were asked, “Are any firearms now kept in or around your home? Do not include firearms that cannot fire. Include those kept in cars or outdoor storage.” If participants indicated the presence of firearms, they were asked about storage practices with 2 follow-up questions. First, participants were asked, “Are any of these firearms now loaded?” If participants responded affirmatively, a second question followed: “Are any of the loaded firearms also unlocked? By ‘unlocked,’ we mean you do not need a key, combination, or hand/fingerprint to get the gun or fire it. Don’t count the safety as a lock.” On the basis of the responses, we categorized firearm storage practices into 3 levels of risk: (1) safest storage practice, where all individuals reported that firearms were stored unloaded; (2) intermediate risk practice, where at least 1 firearm was reported to be both loaded and locked; and (3) unsafe storage practice where at least 1 firearm was reported to be both loaded and unlocked. Respondents with incomplete storage practices data were excluded (n = 18 254) from further analysis.

Firearm regulations vary by state, influencing ownership and storage practices. We analyzed data from 5 states, each with distinct policies on safe storage and child access prevention (CAP) laws. As of 2022, California, Minnesota, and Nevada required locked storage if minors or prohibited individuals could access firearms, whereas New Mexico and Ohio lacked CAP laws.^22,23,24^ Only California mandated locking devices with firearm purchases, whereas Ohio mandates dealers to offer such devices for sale but does not mandate inclusion, as of 2022.^23,25^ Given the potential influence of these policies on firearm storage behaviors, we have adjusted for CAP laws and safe storage requirements in our analysis. We excluded firearm acquisition, background checks, and other restrictions owing to collinearity concerns from the limited state sample. However, we assume CAP laws and safe storage requirements serve as key proxies for policy variation, adequately capturing the impact of regulatory differences on firearm storage practices.

### Social Drivers of Health

The SDOH measures in this study include food insecurity (defined as the respondent reporting always or usually to a question, “During the past 12 months how often did the food that you bought not last, and you didn’t have money to get more?”), housing insecurity (defined as the respondent reporting to have not been able to pay their mortgage, rent or utility bills in past 12 months), social isolation (defined as respondents reporting feeling always or usually socially isolated from others), job loss and/or employment insecurity (defined as the respondent reporting to have lost employment or had reduced hours in past 12 months), feeling stressed (defined as the respondent reporting being always or usually in a situation in which they feels tense, restless, nervous, or anxious, or is unable to sleep at night because their mind is troubled all the time), transportation barriers (defined as the respondent reporting a lack of reliable transportation that kept them from medical appointments, meetings, work, or from getting things needed for daily living in past 12 months), and financial hardship (defined as the respondent reporting to have been threatened by electric, gas, oil or water companies to shut off services in past 12 months). The selection of these measures was supported by their well-documented associations with firearm storage behaviors, injury risk, and disparities in firearm-related harm.^1,9,13,15,18,26,27,28,29^ Although the terms *social drivers of health* and *social determinants of health* are often used interchangeably, the term *determinants* may inadvertently imply that health outcomes are predetermined and unmodifiable.^30^ This framing risks minimizing the accountability of policymakers and decision-makers in addressing social inequities.^31^ Therefore, in this study, we refer to these factors as social *drivers* of health to emphasize the dynamic and modifiable nature of these conditions. To assess the cumulative association of adverse SDOH, we further created a composite SDOH measure by summing the nonmissing (n = 32 526) indicators, resulting in a score ranging from 0 to 7. This composite approach aligns with preexisting public health metrics, which assess SDOH factors comprehensively in order to develop an overarching reflection of community health, as opposed to measuring individual SDOH risk factors.^32,33,34^ The composite SDOH score was categorized into a 4-level ordinal variable: no adverse SDOH (score of 0), 1, 2, and 3 or more adverse SDOH present.

### Statistical Analysis

Data analysis occurred from April 1 to October 2, 2024. We conducted descriptive analyses to estimate the prevalence of firearm ownership and storage practices across subgroups and used χ^2^ tests to evaluate statistically significant differences between groups, with significance set at 2-sided *P* < .05. Using survey commands and CDC-provided weights, we accounted for nonresponse and selection probability to ensure state-representative estimates. To examine the associations between SDOH factors and storage practices, we used separate logistic regression model for each of SDOH factor as a primary exposure. The models estimated adjusted odds ratios (aORs) and 95% CIs, comparing unsafe and intermediate storage practices with safest practice. Covariates were selected a priori, including demographic (age, race and ethnicity, sex, household income, education, marital status, veteran status, and having at least 1 child aged 18 years or younger in a home), behavioral and mental health indicators (binge alcohol use [ie, men having ≥5 drinks on 1 occasion and women having ≥4 drinks on 1 occasion], chronic alcohol use [ie, men having >14 drinks per week and women having >7 drinks per week], cigarette use, poor sleep [ie, <5 hours of sleep on average in 24-hour period], history of depression [ie, a history of depressive disorder, including depression, major depression, dysthymia, or minor depression], life dissatisfaction, frequent mental distress [ie, ≥14 days of unwell mental health days in past month], and physical distress [ie, ≥14 days of unwell physical health days in past month]), and firearm policies (CAP and safe storage), as potential confounders and considered as potential sources of selection bias.

Data on race and ethnicity were obtained from the BRFSS and were classified as American Indian, Alaska Native, and Pacific Islander; non-Hispanic Asian (hereafter, Asian); non-Hispanic Black (hereafter, Black); Hispanic; non-Hispanic White (hereafter, White); and multiracial. Race and ethnicity are included in this study because structural inequities shape differential exposure to SDOH that may influence firearm storage, with evidence showing racial disparities in firearm-related injuries.^1,5,19^

We assessed potential effect measure modification by household income through sensitivity analyses with interaction terms between each SDOH factor and household income in regression models for firearm ownership and storage practices. Because no statistically significant interactions were detected, we reported findings from models without interaction terms. To explore the factors associated with firearm ownership vs no firearm ownership, we conducted a logistic regression model. For storage practices, we ran separate models comparing unsafe and intermediate vs safe storage, adjusting for the same covariates. All statistical analysis was conducted using Stata statistical software version 18.1 (StataCorp).

## Results

The overall study sample included 44 736 adults across 5 states. The weighted population was 52.5% female (95% CI, 51.5%-53.5%); 1.6% (95% CI, 1.4%-1.9%) American Indian, Alaska Native, and Pacific Islander; 10.0% Asian (95% CI, 9.2%-10.8%); 7.7% Black (95% CI, 7.2%-8.3%); 25.1% Hispanic (95% CI, 24.1%-26.1%); 52.5% White (95% CI, 51.5%-53.5%); and 3.1% (95% CI, 2.7%-3.6%) multiracial. Baseline characteristics of participants by firearm ownership are shown in Table 1. Among the weighted population, 70.7% (27 590 participants) were from non–firearm-owning households, and 29.3% (17 146 participants) were from firearm-owning households. Table 2 reports firearm storage practice among firearm owners. Overall, 67.9% (11 396 participants) stored firearms unloaded, 15.9% (2391 participants) stored them loaded and locked, and 16.4% (2816 participants) stored them loaded and unlocked.

**Table 1.** Descriptive Characteristics of Adults by Firearm Ownership and Storage Practice, 2022 Behavioral Risk Factor Surveillance System

Characteristic	Weighted % (95% CI)^a^			*P* value^b^
	Non–firearm-owning households (n = 27 590 [70.7%])	Firearm-owning households (n = 17 146 [29.3%])	Total (N = 44 736 [100.0%])	
Age group, y				
18-24	12.5 (11.6-13.4)	10.5 (9.3-11.7)	11.9 (11.2-12.6)	<.001
25-34	17.6 (16.6-18.6)	15.9 (14.6-17.3)	17.1 (16.3-17.9)	
35-44	16.9 (15.9-17.8)	15.5 (14.3-16.9)	16.5 (15.7-17.3)	
45-54	15.1 (14.2-16.0)	14.9 (13.9-16.1)	15.0 (14.3-15.8)	
55-64	15.8 (14.9-16.8)	17.8 (16.6-19.1)	16.4 (15.7-17.2)	
≥65	22.2 (21.3-23.2)	25.4 (23.9-26.8)	23.1 (22.4-23.9)	
Sex				
Female	56.6 (55.3-57.8)	42.7 (41.0-44.4)	52.5 (51.5-53.5)	<.001
Male	43.4 (42.2-44.7)	57.3 (55.6-59.0)	47.5 (46.5-48.5)	
Race				
American Indian, Alaska Native, and Pacific Islander, non-Hispanic	1.6 (1.3-1.8)	1.9 (1.3-2.6)	1.6 (1.4-1.9)	<.001
Asian, non-Hispanic	12.3 (11.2-13.4)	4.5 (3.5-5.7)	10.0 (9.2-10.8)	
Black, non-Hispanic	8.0 (7.4-8.7)	6.9 (6.0-8.0)	7.7 (7.2-8.3)	
Hispanic	29.6 (28.4-30.8)	14.2 (12.6-15.8)	25.1 (24.1-26.1)	
White, non-Hispanic	45.7 (44.6-46.9)	68.9 (66.9-70.8)	52.5 (51.5-53.5)	
Multiracial	2.9 (2.4-3.4)	3.7 (2.9-4.7)	3.1 (2.7-3.6)	
Education				
Less than high school	15.7 (14.5-16.8)	6.9 (5.7-8.3)	13.1 (12.2-14.0)	<.001
High school graduate	24.6 (23.5-25.6)	29.0 (27.4-30.6)	25.9 (25.0-26.8)	
Some college	29.1 (27.9-30.2)	35.3 (33.7-37.0)	30.9 (30.0-31.9)	
College graduate	30.7 (29.7-31.7)	28.8 (27.4-30.2)	30.1 (29.3-31.0)	
Annual household income, $				
<25 000	20.0 (18.9-21.2)	8.1 (7.1-9.2)	16.3 (15.5-17.2)	<.001
25 000 to <50 000	27.5 (26.3-28.8)	22.2 (20.7-23.9)	25.9 (24.9-26.9)	
50 000 to <100 000	24.5 (23.4-25.7)	32.0 (30.4-33.7)	26.8 (25.9-27.8)	
≥100 000	28.0 (26.7-29.2)	37.6 (35.8-39.4)	30.9 (29.9-32.0)	
Marital status				
Married	44.3 (43.1-45.6)	57.8 (56.1-59.5)	48.3 (47.3-49.3)	<.001
Unmarried	34.9 (33.7-36.1)	26.4 (24.8-28.1)	32.4 (31.4-33.4)	
Divorced, widowed, or separated	20.8 (19.9-21.8)	15.8 (14.7-16.9)	19.3 (18.6-20.1)	
Veteran	5.9 (5.4-6.4)	15.1 (13.9-16.5)	8.6 (8.1-9.2)	<.001
At least 1 child in house	33.5 (32.3-34.8)	34.0 (32.4-35.8)	33.7 (32.7-34.7)	.62
Binge alcohol use^c^	15.7 (14.7-16.6)	21.8 (20.3-23.3)	17.5 (16.7-18.3)	<.001
Chronic alcohol use^c^	5.6 (5.1-6.2)	8.2 (7.3-9.3)	6.4 (5.9-6.9)	<.001
Current cigarette use	11.9 (11.1-12.8)	14.5 (13.3-15.8)	12.7 (12.0-13.4)	<.001
Poor sleep^c^	4.1 (3.7-4.6)	4.2 (3.6-4.9)	4.2 (3.8-4.6)	.87
Depression^c^	22.2 (21.2-23.2)	21.0 (19.7-22.4)	21.9 (21.1-22.7)	.18
Life dissatisfaction	7.1 (6.4-7.8)	5.8 (4.9-6.8)	6.7 (6.2-7.3)	.03
Frequent mental distress^c^	16.1 (15.2-17.0)	15.9 (14.6-17.3)	16.0 (15.3-16.8)	.86
Frequent physical distress^c^	12.9 (12.1-13.8)	12.4 (11.2-13.6)	12.8 (12.1-13.4)	.47
Child access prevention law mandated	70.1 (69.3-70.9)	51.6 (50.1-53.2)	64.7 (64.1-65.3)	<.001
Firearm safe storage mandated	13.8 (13.5-14.2)	21.0 (20.1-21.8)	15.9 (15.6-16.2)	<.001
SDOH^c^				<.001
Food insecurity	4.9 (4.3-5.6)	3.3 (2.5-4.3)	4.5 (4.0-5.0)	.009
Housing insecurity	14.2 (13.2-15.2)	8.3 (7.2-9.4)	12.6 (11.8-13.3)	<.001
Social isolation	10.1 (9.3-11.0)	8.7 (7.5-10.0)	9.7 (9.1-10.4)	.06
Job loss or employment insecurity	16.3 (15.2-17.5)	12.3 (10.9-13.8)	15.2 (14.3-16.1)	<.001
Transportation barriers	14.3 (13.4-15.3)	14.1 (12.7-15.6)	14.3 (13.5-15.1)	<.001
Feeling stressful	9.8 (9.0-10.7)	5.7 (4.7-7.0)	8.7 (8.1-9.4)	.79
Financial hardship	6.8 (6.2-7.5)	6.9 (5.8-8.2)	6.8 (6.3-7.5)	.94
Adverse SDOH composite measure				
None	60.2 (58.8-61.5)	68.1 (66.2-69.9)	62.3 (61.2-63.4)	<.001
1 Adverse SDOH present	21.0 (19.8-22.2)	18.0 (16.6-19.6)	20.2 (19.3-21.2)	
2 Adverse SDOH present	9.1 (8.4-9.9)	6.9 (6.0-8.1)	8.5 (7.9-9.2)	
≥3 Adverse SDOH present	9.7 (8.9-10.5)	6.9 (5.9-8.2)	9.0 (8.3-9.6)	

Abbreviation: SDOH, social drivers of health.

^a^ The overall numbers for the sample by groups are reported with unweighted numbers and weighted percentages.

^b^ χ^2^ tests were used to assess differences in characteristics by firearm ownership, with *P* < .05 level of significance.

^c^ Defined in the Methods section.

**Table 2.** Descriptive Characteristics of Firearm Owning Adults by Storage Practices, 2022 Behavioral Risk Factor Surveillance System

Characteristic	Weighted % (95% CI) (N = 16 603)^a^			*P* value^b^
	Safest storage practice: unloaded (n = 11 396 [67.9%])	Intermediate risk: loaded and locked (n = 2391 [15.9%])	Unsafe storage practice: loaded and unlocked (n = 2816 [16.4%])	
Age group, y				
18-24	11.3 (9.8-12.8)	7.9 (5.9-10.6)	6.3 (4.6-8.6)	<.001
25-34	15.4 (13.9-17.0)	17.1 (13.7-21.2)	17.7 (13.8-22.5)	
35-44	15.9 (14.3-17.7)	19.2 (16.1-22.6)	11.7 (9.1-14.9)	
45-54	13.8 (12.6-15.2)	19.1 (16.1-22.4)	16.2 (13.5-19.4)	
55-64	18.3 (16.8-19.9)	17.2 (14.3-20.6)	17.3 (14.5-20.5)	
≥65	25.4 (23.6-27.2)	19.5 (16.6-22.8)	30.8 (26.8-35.0)	
Sex				
Female	46.9 (44.8-49.1)	33.7 (30.0-37.6)	29.1 (25.0-33.6)	<.001
Male	53.1 (50.9-55.2)	66.3 (62.4-70.0)	70.9 (66.4-75.0)	
Race				
American Indian, Alaska Native, and Pacific Islander, non-Hispanic	2.0 (1.3-3.2)	1.9 (1.0-3.8)	1.4 (0.8-2.5)	<.001
Asian, non-Hispanic	4.5 (3.4-6.0)	4.8 (2.4-9.1)	3.8 (1.5-9.4)	
Black, non-Hispanic	5.1 (4.1-6.2)	11.9 (9.0-15.7)	9.8 (7.3-13.0)	
Hispanic	15.5 (13.6-17.7)	9.4 (7.3-12.1)	11.9 (8.5-16.4)	
White, non-Hispanic	69.9 (67.5-72.2)	67.8 (63.1-72.2)	67.3 (62.0-72.2)	
Multiracial	3 (2.1-4.1)	4.1 (2.5-6.8)	5.8 (3.5-9.5)	
Education				
Less than high school	7.8 (6.2-9.9)	4.1 (2.7-6.1)	6.0 (4.1-8.7)	<.001
High school graduate	27.8 (25.9-29.7)	28.2 (24.4-32.5)	35.4 (31.0-40.1)	
Some college	33.1 (31.1-35.1)	39.5 (35.4-43.7)	38.7 (34.3-43.2)	
College graduate	31.3 (29.6-33.1)	28.2 (24.9-31.7)	19.9 (17.4-22.8)	
Annual household income, $				
<25 000	7.9 (6.7-9.3)	6.1 (4.6-8.0)	10.7 (7.7-14.8)	.19
25 000 to <50 000	22.5 (20.5-24.6)	21.0 (17.8-24.8)	23.6 (20.1-27.5)	
50 000 to <100 000	31.9 (29.9-34.0)	33.8 (29.6-38.3)	30.6 (26.8-34.6)	
≥100 000	37.7 (35.5-39.9)	39.1 (35.0-43.3)	35.1 (30.4-40.0)	
Marital status				
Married	59.6 (57.5-61.7)	58.2 (53.9-62.3)	52.1 (47.6-56.6)	<.001
Unmarried	26.1 (24.1-28.1)	25.8 (21.8-30.3)	25.5 (21.6-29.8)	
Divorced, widowed, or separated	14.3 (13.0-15.7)	16.0 (13.7-18.5)	22.4 (19.4-25.8)	
Veteran	13.0 (11.5-14.6)	20.0 (16.2-24.4)	21.3 (17.9-25.1)	<.001
At least 1 child in house	36.0 (33.9-38.1)	41.5 (37.4-45.7)	21.2 (17.1-26.0)	<.001
Binge alcohol use^c^	20.3 (18.6-22.2)	22.1 (19.1-25.4)	26.7 (22.4-31.4)	.01
Chronic alcohol use^c^	7.3 (6.2-8.7)	8.8 (6.9-11.2)	10.3 (7.6-13.8)	.08
Current cigarette use	13.0 (11.6-14.6)	17.9 (14.7-21.7)	18.3 (15.2-21.8)	.001
Poor sleep^c^	3.5 (2.8-4.4)	7.2 (5.5-9.4)	5.0 (3.6-6.9)	<.001
Depression^c^	20.5 (18.9-22.1)	20.5 (17.1-24.4)	20.7 (17.2-24.6)	>.99
Life dissatisfaction	5.0 (4.1-6.1)	5.4 (3.6-7.8)	9.1 (5.8-13.8)	.02
Frequent mental distress^c^	14.8 (13.3-16.5)	21.4 (17.4-26.0)	14.3 (11.8-17.3)	.002
Frequent physical distress^c^	11.4 (10.0-13.0)	13.5 (10.8-16.7)	14.3 (11.7-17.3)	.13
Child access prevention law mandated	55.7 (54.1-57.3)	41.7 (37.7-45.8)	43.3 (38.8-48.0)	<.001
Firearm safe storage mandated	22.0 (21.1-22.9)	18.2 (16.3-20.4)	20.2 (18.1-22.6)	.02
SDOH^c^				
Food insecurity	2.3 (1.6-3.4)	4.0 (2.6-6.1)	6.9 (3.6-12.6)	.004
Housing insecurity	7.1 (6.1-8.4)	9.3 (7.1-12.0)	11.6 (8.0-16.6)	.02
Social isolation	7.5 (6.3-8.9)	11.0 (7.4-16.0)	10.6 (7.3-15.3)	.10
Job loss or employment insecurity	11.7 (10.1-13.5)	13.0 (10.0-16.8)	13.5 (9.7-18.6)	.61
Transportation barriers	13.0 (11.5-14.7)	16.0 (12.3-20.6)	15.9 (11.9-20.9)	.02
Feeling stressful	4.8 (3.7-6.2)	6.6 (4.5-9.6)	7.9 (4.8-12.8)	.11
Financial hardship	5.5 (4.3-6.9)	9.2 (6.7-12.5)	11.9 (7.9-17.6)	.001
Adverse SDOH composite measure				
None	70.2 (68.0-72.4)	63 (58.0-67.8)	63.8 (58.3-69.0)	.04
1 Adverse SDOH present	16.8 (15.2-18.7)	21.7 (17.9-26.1)	19.3 (15.3-24.1)	
2 Adverse SDOH present	7.1 (5.9-8.4)	6.6 (3.8-11.0)	7.3 (5.1-10.3)	
≥3 Adverse SDOH present	5.8 (4.6-7.3)	8.7 (6.2-11.9)	9.6 (6.2-14.5)	

Abbreviation: SDOH, social drivers of health.

^a^ Table does not present information on 543 respondents in firearm-owning households that were missing information on storage practices. The overall numbers for the subgroups are reported with unweighted numbers and weighted percentages.

^b^ χ^2^ tests were used to assess differences in characteristics by firearm ownership, with *P* < .05 level of significance.

^c^ Defined in the Methods section.

Respondents who reported storing firearms unloaded (considered the safest storage practice) were more likely than those who stored firearms loaded and unlocked (an unsafe storage practice) to be younger (aged 18-24 years, 11.3% [95% CI, 9.8%-12.8%] vs 6.3% [95% CI, 4.6%-8.6%]) and female (46.9% [95% CI, 44.8%-49.1%] vs 29.1% [95% CI, 25.0%-33.6%]). Racial disparities were evident, with the safest storage practice more common among White (69.9% [95% CI, 67.5%-72.2%] vs 67.3% [95% CI, 62.0%-72.2%]) and Hispanic (15.5% [95% CI, 13.6%-17.7%] vs 11.9% [95% CI, 8.5%-16.4%]) firearm owners and less common among Black firearm owners (5.1% [95% CI, 4.1%-6.2%] vs 9.8% [95% CI, 7.3%-13.0%]) (eTable 1 in Supplement 1).

Unsafe storage was more common than the safest storage practice among respondents reporting binge alcohol use (26.7% [95% CI, 22.4%-31.4%] vs 20.3% [95% CI, 18.6%-22.2%]), cigarette use (18.3% [95% CI, 15.2%-21.8%] vs 13.0% [95% CI, 11.6%-14.6%]), poor sleep (5.0% [95% CI, 3.6%-6.9%] vs 3.5% [95% CI, 2.8%-4.4%]), and life dissatisfaction (9.1% [95% CI, 5.8%-13.8%] vs 5.0% [95% CI, 4.1%-6.1%]), but it was less common among respondents in states with CAP laws (43.3% [95% CI, 38.8%-48.0%] vs 55.7% [95% CI, 54.1%-57.3%]) and safe storage laws (20.2% [95% CI, 18.1%-22.6%] vs 22.0% [95% CI, 21.1%-22.9%]). For more details, see eTable 2 in Supplement 1.

Table 3 shows aORs for intermediate and unsafe firearm storage vs safe storage practices among firearm owners. Unsafe storage was more likely among those facing food insecurity (aOR, 3.09; 95% CI, 1.29-7.40), housing insecurity (aOR, 1.66; 95% CI, 1.01-2.79), transportation barriers (aOR, 2.16; 95% CI, 1.19-3.90), and financial hardship (aOR, 2.22; 95% CI, 1.16-4.28). The aORs for social isolation (aOR, 1.53; 95% CI, 0.95-2.46) and stress (aOR, 1.64; 95% CI, 0.97-2.78) were increased but not statistically significant. No significant associations were found for intermediate storage; the aOR for stress was decreased (aOR, 0.89; 95% CI, 0.60-1.31) but not statistically significant.

**Table 3.** Associations Between SDOH and Storage Practices Among Firearm-Owning Participants (N = 32 536)

SDOH^a^	aOR (95% CI)	
	Intermediate risk storage (loaded and locked)^b^	Unsafe storage (loaded and unlocked)^b^
Food insecurity	1.78 (0.94-3.38)	3.09 (1.29-7.40)^c^
Housing insecurity	0.97 (0.62-1.51)	1.66 (1.01-2.79)^c^
Social isolation	1.14 (0.69-1.87)	1.53 (0.95-2.46)
Job loss or employment insecurity	0.91 (0.61-1.36)	1.20 (0.81-1.78)
Feeling stressful	0.89 (0.60-1.31)	1.64 (0.97-2.78)
Transportation barriers	1.28 (0.76-2.14)	2.16 (1.19-3.90)^c^
Financial hardship	1.18 (0.73-1.89)	2.22 (1.16-4.28)^c^

Abbreviations: aOR, adjusted odds ratio; SDOH, social drivers of health.

^a^ Defined in the Methods section.

^b^ Logistic regression models were conducted for each SDOH as primary exposure, adjusted for demographics, behavioral factors, mental health factors, and firearm related policies. Safest (unloaded) storage practice is the referent category for aORs.

^c^ Level of confidence, *P* < .05.

Table 4 presents factors associated with firearm ownership and storage practices. Although small or no associations were found between SDOH and firearm ownership, firearm owners with SDOH burdens had higher odds of unsafe storage, especially those with 3 or more SDOH (aOR, 2.06; 95% CI, 1.07-3.97) compared with those with no SDOH burden. Younger individuals (aged 25-54 years) were more likely than older individuals to engage in unsafe or intermediate storage. Female respondents were less likely than male respondents to own firearms (aOR, 0.67; 95% CI, 0.59-0.75) and practice unsafe storage (aOR, 0.59; 95% CI, 0.42-0.83). Black (aOR, 0.72; 95% CI, 0.57-0.91), Asian (aOR, 0.35; 95% CI, 0.25-0.49), and Hispanic (aOR, 0.54; 95% CI, 0.45-0.64) individuals were less likely to own firearms than White individuals. However, Black firearm owners had higher odds of unsafe storage (aOR, 2.23; 95% CI, 1.39-3.57); the aORs for unsafe storage were increased for multiracial (aOR, 1.29; 95% CI, 0.37-4.44) and Hispanic (aOR, 1.28; 95% CI, 0.82-1.99) firearm owners but were not statistically significant. Black owners were also more likely to practice intermediate storage (aOR, 2.56; 95% CI, 1.61-4.07) compared with White individuals.

**Table 4.** Association of Firearm Ownership With Storage Practice (N = 32 536)

Characteristics	aOR (95% CI)		
	Firearm ownership^a^	Intermediate risk (loaded and locked)^b^	Unsafe storage practice (loaded and unlocked)^b^
SDOH composite measure (reference, no SDOH)			
1 SDOH present	0.95 (0.80-1.12)	1.30 (0.95-1.79)	1.52 (1.03-2.23)^c^
2 SDOH present	1.02 (0.79-1.30)	0.72 (0.42-1.27)	1.14 (0.62-2.09)
≥3 SDOH present	0.96 (0.70-1.31)	0.91 (0.51-1.61)	2.06 (1.07-3.97)^c^
Age group, y (reference, 18-24 y)			
25-34	1.13 (0.86-1.48)	1.28 (0.73-2.25)	2.06 (1.11-3.80)^c^
35-44	1.06 (0.80-1.42)	1.13 (0.62-2.04)	1.83 (0.92-3.66)
45-54	1.04 (0.78-1.38)	1.51 (0.84-2.73)	1.77 (0.96-3.24)
55-64	1.34 (0.99-1.81)	0.98 (0.54-1.77)	1.14 (0.60-2.16)
≥65	1.48 (1.09-2.01)^c^	0.79 (0.44-1.43)	1.55 (0.81-2.98)
Female sex (reference, male)	0.67 (0.59-0.75)^c^	0.49 (0.38-0.63)^c^	0.59 (0.42-0.83)^c^
Race (reference, White, non-Hispanic)			
American Indian, Alaska Native, and Pacific Islander, non-Hispanic	1.04 (0.65-1.65)	0.81 (0.34-1.91)	0.75 (0.31-1.82)
Asian, non-Hispanic	0.35 (0.25-0.49)^c^	1.07 (0.49-2.34)	1.29 (0.37-4.44)
Black, non-Hispanic	0.72 (0.57-0.91)^c^	2.56 (1.61-4.07)^c^	2.23 (1.39-3.57)^c^
Hispanic	0.54 (0.45-0.64)^c^	0.81 (0.56-1.16)	1.28 (0.82-1.99)
Multiracial	1.19 (0.80-1.78)	1.14 (0.51-2.56)	1.42 (0.75-2.68)
Education (reference, less than high school)			
High school graduate	1.49 (1.07-2.07)^c^	2.28 (1.16-4.47)^c^	1.99 (1.05-3.77)^c^
Some college	1.33 (0.96-1.86)	2.84 (1.44-5.61)^c^	1.95 (1.06-3.61)^c^
College graduate	0.83 (0.59-1.17)	2.09 (1.06-4.13)^c^	0.95 (0.51-1.76)
Annual household income, $ (reference, <$25 000)			
25 000 to <50 000	1.71 (1.37-2.14)^c^	1.72 (1.08-2.76)^c^	0.89 (0.54-1.46)
50 000 to <100 000	2.57 (2.06-3.20)^c^	1.79 (1.12-2.87)^c^	1.11 (0.70-1.76)
≥100 000	3.34 (2.62-4.24)^c^	1.92 (1.17-3.13)^c^	1.35 (0.81-2.27)
Marital status (reference, married)			
Unmarried	0.80 (0.67-0.96)^c^	0.82 (0.57-1.19)	0.80 (0.54-1.19)
Divorced, widowed, or separated	0.68 (0.58-0.79)^c^	1.11 (0.83-1.49)	1.51 (1.11-2.07)^c^
Veteran (reference, civilian)	1.97 (1.58-2.45)^c^	1.40 (1.00-1.97)	1.54 (1.12-2.13)^c^
At least 1 child in house (reference, no child in house)	1.22 (1.05-1.43)^c^	1.05 (0.80-1.39)	0.38 (0.26-0.55)^c^
Binge alcohol use (reference, no binge alcohol use)	1.34 (1.14-1.58)^c^	0.87 (0.65-1.17)	1.39 (1.01-1.93)^c^
Chronic alcohol use (reference, no chronic alcohol use)	1.22 (0.93-1.61)	1.56 (1.02-2.40)^c^	1.12 (0.70-1.82)
Current cigarette use (reference, no current cigarette use)	1.13 (0.94-1.36)	1.52 (1.08-2.14)^c^	1.36 (0.97-1.89)
Poor sleep (reference, adequate sleep)	1.11 (0.83-1.49)	1.48 (0.90-2.44)	0.83 (0.48-1.46)
Depression (reference, no history of depression)	0.88 (0.75-1.02)	1.00 (0.72-1.38)	1.04 (0.76-1.44)
Life dissatisfaction (reference, life satisfaction)	1.10 (0.83-1.46)	0.85 (0.48-1.50)	1.81 (1.05-3.11)^c^
Frequent mental distress (reference, no frequent mental distress)	1.17 (0.94-1.45)	1.65 (1.13-2.39)^c^	0.61 (0.40-0.93)^c^
Frequent physical distress (reference, no frequent physical distress)	1.02 (0.82-1.27)	1.34 (0.90-1.99)	1.28 (0.88-1.88)
Child access prevention laws mandated (reference, no laws)	0.57 (0.51-0.63)^c^	0.58 (0.45-0.76)^c^	0.52 (0.40-0.68)^c^
Firearm safe storage laws mandated (reference, no safe storage laws)	1.76 (1.60-1.94)^c^	0.95 (0.75-1.19)	1.25 (0.98-1.59)

Abbreviations: aOR, adjusted odds ratio; SDOH, social drivers of health.

^a^ Non–firearm-owning household is the referent category for aORs.

^b^ Unloaded storage is the referent category for aORs.

^c^ Level of significance, *P* < .05.

Individuals with a high school education had a higher likelihood of firearm ownership (aOR, 1.49; 95% CI, 1.07-2.07) and unsafe storage practice (aOR, 1.99; 95% CI, 1.05-3.77) than those with less than high school education. Income was a factor associated with ownership, with those earning $100 000 or more having more than 3 times the odds of owning a firearm compared with those earning less than $25 000 (aOR, 3.34; 95% CI, 2.62-4.24). Higher income was associated with storing firearms locked and loaded but not with unsafe storage. Compared with married individuals, divorced, widowed, or separated individuals had lower odds of firearm ownership (aOR, 0.68; 95% CI, 0.58-0.79) but higher odds of unsafe storage (aOR, 1.51; 95% CI, 1.11-2.07).

Households with children had higher firearm ownership odds (aOR, 1.22; 95% CI, 1.05-1.43) but lower unsafe storage odds (aOR, 0.38; 95% CI, 0.26-0.55) compared with households without any children. Compared with households residing in states without CAP law, the odds of firearm ownership (aOR, 0.57; 95% CI, 0.51-0.63) and unsafe storage (aOR, 0.52; 95% CI, 0.40-0.68) were lower in households from states with CAP laws. Safe storage policies in states increased the likelihood of firearm ownership (aOR, 1.76; 95% CI, 1.60-1.94) but were not associated with unsafe storage (aOR, 1.25; 95% CI, 0.98-1.59).

Other factors associated with unsafe storage practices included veteran status (aOR, 1.54; 95% CI, 1.12-2.13), binge alcohol use (aOR, 1.39; 95% CI, 1.01-1.93), and life dissatisfaction (aOR, 1.81; 95% CI, 1.05-3.11). A negative association with frequent mental distress (aOR, 0.61; 95% CI, 0.40-0.93) was also observed for unsafe storage practice.

## Discussion

To our knowledge, this cross-sectional study is one of the first to explore the association between firearm ownership, storage practices, and SDOH using data from a representative sample via BRFSS. The findings reveal that firearm ownership is more prevalent among White households, particularly those with higher education and income levels. In contrast, unsafe firearm storage is more frequently reported in Black households and those with lower education and income. The likelihood of unsafe storage significantly increases in firearm owners experiencing SDOH burdens, such as food insecurity, housing insecurity, financial instability, and transportation challenges.

We found significant associations between socioeconomic status, firearm ownership, and storage practices. Firearm ownership was the most prevalent among higher-income groups (≥$100 000), although unsafe storage was more frequent among individuals with lower incomes. Prior research suggests that firearm ownership in US is closely tied to socioeconomic status, reflecting cultural traditions rooted in a desire to protect personal assets from a centralized authority.^35^ Historically, the largest group of firearm owners has been older White men,^17^ as evident in this study as well, who also tend to have the greatest wealth accumulation.^36^ However, ownership has diversified substantially in recent years; between 2020 and 2022, 89% of first-time firearm owners were younger than 45 years, and 69% were from minoritized racial and ethnic groups.^37^

Beyond ownership, firearm storage practices are also influenced by socioeconomic factors, particularly financial stability. We found greater likelihood of unsafe storage practice in the presence of financial hardship among firearm owners. Previous literature suggests that households with lower socioeconomic status often cite the cost of safety devices as a barrier to secure storage.^18^ Although financial hardship may lead to prioritizing immediate needs like food, rent, and utilities over safety measure, the financial stress may also contribute to a heightened perception of personal insecurity,^16^ leading some individuals to keep firearms loaded and accessible for perceived self-defense.

We found that firearm ownership is more common among White adults, whereas unsafe storage practices are prevalent among Black individuals. During COVID-19, firearm ownership nearly doubled, with notable increases among Black and Asian individuals, largely due to concerns about racial violence.^28,38,39^ Studies show that 36% of Black and 42% of Asian individuals cited racial violence as a key reason for firearm purchases.^38^ Black communities also face disproportionately higher rates of firearm violence, including homicide, which may influence storage behaviors that prioritize quick access for self-defense.^40,41,42^ Moreover, systemic and socioeconomic factors, such as economic constraints, may limit access to secure storage options like safes or lockboxes. Addressing these disparities requires targeted interventions that account for the interplay of SDOH and firearm safety behaviors to reduce risks associated with unsafe storage and gun violence.

Depression showed no link to unsafe storage, whereas frequent mental distress was associated with safer storage and life dissatisfaction was associated with unsafe storage. These findings challenge the *dangerous people* framework, which oversimplifies gun violence by overlooking broader social drivers.^43^ Shifting focus from individual mental health to systemic socioeconomic factors is key to understanding firearm safety behaviors and addressing root causes of risk.

We found lower odds of unsafe firearm storage in homes with children; however, firearm ownership was more common, presenting a critical public health issue in the context of school shootings. Policies aimed at reducing child access to firearms must account for behavioral risk factors of both parents and children that influence storage practices. Furthermore, we found lower odds of firearm ownership and unsafe storage in states with CAP laws. Evidence suggests that CAP laws that impose liability on firearm owners for unsafe storage practices are linked to greater reductions in child firearm fatalities compared with CAP laws that solely penalize the direct provision of firearms to children.^44^ The variability in state-level firearm policies highlights the fragmented nature of US firearm regulations and underscores the need for policy reform to promote safer firearm storage practices.

Policy should address SDOH as upstream forces behind firearm deaths, recognizing their role in mental distress and behavioral risks. Amid the US Surgeon General’s declaration of firearm violence as a public health crisis, solutions must go beyond mental health interventions to tackle financial insecurity and structural racism.^1^ These findings highlight the limitations of gun buyback programs, which reduce firearm numbers but have minimal impact on firearm violence.^45^ Research indicates that high-risk individuals may avoid buybacks due to distrust of law enforcement and reliance on firearms for self-defense in the context of perceived police ineffectiveness,^45,46,47,48^ both of which are linked to structural and symbolic racism.^46^ Participation is most likely when the benefits of turning in a firearm outweigh the perceived costs. Addressing the underlying social risks may help shift this balance by reducing perceived costs, ultimately decreasing firearm availability and potentially mitigating both self-inflicted and community violence.

### Limitations

This analysis has several limitations. First, only 5 states implemented the firearm safety and SDOH modules in 2021 or 2022, limiting the generalizability of findings. Selection bias may have occurred, as participants practicing safer storage or those without firearms may have been more likely to respond, potentially underestimating our results. However, survey weighting and covariate adjustments likely mitigated this bias. Second, the self-reported nature of BRFSS data introduces other potential biases, such as social desirability and recall issues, particularly regarding firearm presence and storage practices. Third, although we accounted for relevant firearm safety and storage policies, our analysis was limited to selected states and specific policies, which may not fully capture their effects. Despite these limitations, our findings provide valuable insights into firearm storage behaviors and associated risks for guiding efforts to promote secure storage and reduce firearm-related harm.

## Conclusions

The study identified firearm ownership as more prevalent among White households, as well as those with higher education and income levels. Conversely, unsafe firearm storage was more commonly observed in Black households and those with lower education and income. SDOH, such as food, housing, and financial insecurity, and transportation barriers, along with high-risk behaviors like alcohol misuse and life dissatisfaction, were more prevalent in firearm-owning households with unsafe storage. Addressing these disparities with targeted public health initiatives and education is critical for enhancing firearm safety.

## Notes

Supplement 1. **eTable 1.** Associations Between Social Drivers of Health and Intermediate risk (Loaded and Locked) Storage Practice Among Firearm-Owning Participants **eTable 2.** Associations Between Social Drivers of Health and Unsafe (Loaded and Unlocked) Storage Practice Among Firearm-Owning Participants

Supplement 2.
Data Sharing Statement
