# Supplementary material for: Social Drivers of Health and Firearm Storage Practices
Source: JAMA Netw Open. 2025 Jun 2;8(6):e2513280. doi: 10.1001/jamanetworkopen.2025.13280 (PMC12131096; doi:10.1001/jamanetworkopen.2025.13280)
Supplement: Supplement 1. — eTable 1. Associations Between Social Drivers of Health and Intermediate risk (Loaded and Locked) Storage Practice Among Firearm-Owning Participants eTable 2. Associations Between Social Drivers of Health and Unsafe (Loaded and Unlocked) Storage Practice Among Firearm-Owning Participants [file jamanetwopen-e2513280-s001.pdf]

Supplemental Online Content

Parekh T, Pena A, Bhaskar M, Park JW. Social drivers of health and firearm storage practices. *JAMA Netw Open*. 2025;8(6):e2513280.
doi:10.1001/jamanetworkopen.2025.13280

eTable 1. Associations Between Social Drivers of Health and Intermediate risk (Loaded and Locked) Storage Practice Among Firearm-Owning Participants

eTable 2. Associations Between Social Drivers of Health and Unsafe (Loaded and Unlocked) Storage Practice Among Firearm-Owning Participants

This supplemental material has been provided by the authors to give readers additional information about their work.

© 2025 Parekh T et al. *JAMA Network Open*.

eTable 1. Associations Between Social Drivers of Health and Intermediate risk (Loaded and Locked) Storage Practice Among Firearm-Owning Participants

Social drivers of health	Model-0	Model-1	Model-2	Model-3	Model-4
	**OR (95% CI)**	**OR (95% CI)**	**OR (95% CI)**	**OR (95% CI)**	**OR (95% CI)**
Food insecurity	1.79 (0.98 - 3.29)	1.87* (1.01 - 3.47)	1.94* (1.01 - 3.72)	1.78 (0.94 - 3.38)	1.78 (0.94 - 3.38)
Housing insecurity	1.34 (0.95 - 1.87)	1.14 (0.79 - 1.66)	1.08 (0.72 - 1.63)	0.96 (0.62 - 1.50)	0.97 (0.62 - 1.51)
Social isolation	1.53 (0.96 - 2.45)	1.50* (1.01 - 2.24)	1.33 (0.83 - 2.14)	1.15 (0.71 - 1.87)	1.14 (0.69 - 1.87)
Job loss/employment insecurity	1.09 (0.77 - 1.54)	1.02 (0.72 - 1.46)	0.96 (0.66 - 1.38)	0.9 (0.61 - 1.34)	0.91 (0.61 - 1.36)
Feeling stressful	1.25 (0.89 - 1.76)	1.14 (0.83 - 1.59)	1.18 (0.82 - 1.68)	0.89 (0.60 - 1.31)	0.89 (0.60 - 1.31)
Transportation barriers	1.36 (0.83 - 2.24)	1.43 (0.92 - 2.23)	1.39 (0.85 - 2.27)	1.24 (0.74 - 2.08)	1.28 (0.76 - 2.14)
Financial hardship	1.69* (1.10 - 2.58)	1.49 (0.98 - 2.27)	1.33 (0.84 - 2.10)	1.2 (0.75 - 1.93)	1.18 (0.73 - 1.89)

Note:

Model-0: Unadjusted
Model-1: Adjusted for demographics (age, gender, race/ethnicity, education, marital status, veteran status)
Model-2: Adjusted for model-1 + at least one child in a house, and household income
Model-3: Adjusted for model-2 + behavioral and mental health indicators (binge drinking, heavy drinking, current smoking, poor sleep, depression history, life dissatisfaction, frequent mental and physical distress)
Model-4: Adjusted for model-3 + CAP laws in respondents' states, and safe storage laws in respondents' states.

OR: Odds Ratio; CI: Confidence interval;
* Level of confidence, $p<0.05$
[a] Logistic regression models were conducted for each SDOH as primary exposure, with reference category as unlocked storage practice for ORs.

Definition:

- **Food insecurity:** Respondent reporting always or usually to a question "During the past 12 months how often did the food that you bought not last, and you didn't have money to get more?
- **Housing insecurity:** Respondent reporting to have not been able to pay his/her mortgage, rent or utility bills in past 12 months.
- **Social isolation:** Respondents reporting feeling always or usually socially isolated from others.
- **Job loss/Employment insecurity**: Respondent reporting to have lost employment or had reduced hours in past 12 months.
- **Feeling stressed:** Respondent reporting being always or usually in a situation in which he/she feels tense, restless, nervous, or anxious, or is unable to sleep at night because his/her mind is troubled all the time
- **Transportation barriers:** Respondent reporting a lack of reliable transportation that kept him/her from medical appointments, meetings, work, or from getting things needed for daily living in past 12 months.
- **Financial hardship:** Respondent reporting to have been threatened by electric, gas, oil or water companies to shut off services in past 12 months.

© 2025 Parekh T et al. *JAMA Network Open*.

eTable 2. Associations Between Social Drivers of Health and Unsafe (Loaded and Unlocked) Storage Practice Among Firearm-Owning Participants

Social drivers of health	Model-0	Model-1	Model-2	Model-3	Model-4
	**OR (95% CI)**	**OR (95% CI)**	**OR (95% CI)**	**OR (95% CI)**	**OR (95% CI)**
Food insecurity	3.11* (1.39 - 6.95)	3.06* (1.45 - 6.45)	3.46* (1.58 - 7.56)	2.81* (1.21 - 6.54)	3.09* (1.29 - 7.40)
Housing insecurity	1.74* (1.10 - 2.75)	1.53 (0.96 - 2.42)	1.62 (1.00 - 2.61)	1.56 (0.95 - 2.59)	1.66* (1.01 - 2.79)
Social isolation	1.44 (0.90 - 2.29)	1.44 (0.93 - 2.25)	1.59* (1.01 - 2.52)	1.53 (0.96 - 2.43)	1.53 (0.95 - 2.46)
Job loss/employment insecurity	1.15 (0.75 - 1.75)	1.17 (0.79 - 1.71)	1.2 (0.81 - 1.78)	1.16 (0.79 - 1.71)	1.2 (0.81 - 1.78)
Feeling stressful	1.27 (0.88 - 1.84)	1.43 (0.97 - 2.12)	1.51 (1.00 - 2.28)	1.53 (0.92 - 2.56)	1.64 (0.97 - 2.78)
Transportation barriers	1.73 (0.93 - 3.20)	1.95* (1.12 - 3.39)	2.09* (1.17 - 3.74)	2.01* (1.12 - 3.62)	2.16* (1.19 - 3.90)
Financial hardship	2.36* (1.40 - 4.00)	2.21* (1.27 - 3.86)	2.27* (1.23 - 4.21)	2.18* (1.13 - 4.18)	2.22* (1.16 - 4.28)

Note:

Model-0: Unadjusted
Model-1: Adjusted for demographics (age, gender, race/ethnicity, education, marital status, veteran status)
Model-2: Adjusted for model-1 + at least one child in a house, and household income
Model-3: Adjusted for model-2 + behavioral and mental health indicators (binge drinking, heavy drinking, current smoking, poor sleep, depression history, life dissatisfaction, frequent mental and physical distress)
Model-4: Adjusted for model-3 + CAP laws in respondents' states, and safe storage laws in respondents' states.

OR: Odds Ratio; CI: Confidence interval;
* Level of confidence, $p<0.05$
[a] Logistic regression models were conducted for each SDOH as primary exposure, with reference category as unlocked storage practice for ORs.

Definition:

- **Food insecurity:** Respondent reporting always or usually to a question "During the past 12 months how often did the food that you bought not last, and you didn't have money to get more?
- **Housing insecurity:** Respondent reporting to have not been able to pay his/her mortgage, rent or utility bills in past 12 months.
- **Social isolation:** Respondents reporting feeling always or usually socially isolated from others.
- **Job loss/Employment insecurity**: Respondent reporting to have lost employment or had reduced hours in past 12 months.
- **Feeling stressed:** Respondent reporting being always or usually in a situation in which he/she feels tense, restless, nervous, or anxious, or is unable to sleep at night because his/her mind is troubled all the time
- **Transportation barriers:** Respondent reporting a lack of reliable transportation that kept him/her from medical appointments, meetings, work, or from getting things needed for daily living in past 12 months.
- **Financial hardship:** Respondent reporting to have been threatened by electric, gas, oil or water companies to shut off services in past 12 months.

© 2025 Parekh T et al. *JAMA Network Open*.
