# Supplementary material for: Social Drivers of Health and Firearm Storage Practices
Source: JAMA Netw Open. 2025 Jun 2;8(6):e2513280. doi: 10.1001/jamanetworkopen.2025.13280 (PMC12131096; doi:10.1001/jamanetworkopen.2025.13280)
Supplement: Supplement 2. — Data Sharing Statement [file jamanetwopen-e2513280-s002.pdf]

Data Sharing Statement

Parekh. Social Drivers of Health and Firearm Storage Practices. *JAMA Netw Open*. Published June 02, 2025. doi:10.1001/jamanetworkopen.2025.13280

Data
Data available: No

Additional Information
Explanation for why data not available: Data is publicly available. link provided in the manuscript.
